# Analysis of the Rheological Properties of Natural Hydraulic Lime-Based Suspensions for Sustainable Construction and Heritage Conservation

**DOI:** 10.3390/ma17040825

**Published:** 2024-02-08

**Authors:** Ángel De La Rosa, Gonzalo Ruiz, Rodrigo Moreno

**Affiliations:** 1 ETS de Ingenieros de Caminos, C. y P., Universidad de Castilla–La Mancha, Av. Camilo José Cela 2, 13071 Ciudad Real, Spain; angel.delarosa@uclm.es (Á.D.L.R.); gonzalo.ruiz@uclm.es (G.R.); 2 Instituto de Cerámica y Vidrio (CSIC), C. Kelsen 5, Campus de Cantoblanco, 28049 Madrid, Spain

**Keywords:** natural hydraulic lime-based suspension, processing technology, rheology, sustainable construction, heritage conservation

## Abstract

Natural hydraulic lime (NHL)-based binders play a crucial role in preserving cultural heritage structures, ensuring integrity and longevity. Beyond traditional uses, these binders exhibit potential for integration into both non-structural and structural components, being compatible with innovative manufacturing processes such as digital fabrication. Meticulously designed grouts, with applicability in their fresh and hardened states, are essential for heritage stability. This study explores the relationships between mineral additions, chemical admixtures, and lime for grout formulations, aiming to advance our understanding and inform the optimization of materials for heritage restoration. Key questions include the influence of natural volcanic pozzolan (NVP) and metakaolin (MK) on rheology and the impact of varying ratios of superplasticizer on NHL-based grout’s rheological behavior. This systematic evaluation of rheological parameters aims to innovate mix designs, expanding NHL-based binders’ applicability in construction and science. Our hypotheses suggest that well-designed lime grout formulations, incorporating NVP and MK, can enhance rheological properties, addressing challenges in sustainable construction and heritage conservation. This research provides valuable insights for optimizing lime-based materials, fostering advancements in heritage restoration, and promoting wider NHL-based binder adoption in diverse construction applications.

## 1. Introduction

The widespread use of natural hydraulic lime grout in the conservation and rehabilitation of historical structures represents a technique that involves the pressurized injection of a fluid suspension into voids and cracks. This method, recognized for its efficacy in enhancing structural integrity, has become a cornerstone in heritage maintenance and restoration projects [1,2,3]. The utility of lime grout in these applications is rooted in its ability to address key considerations such as injectability, bleed and shrinkage stability, wettability, mechanical properties, and substrate compatibility, especially in terms of mechanical bonding [1,4].

The contemporary preference for lime-based grouts in heritage preservation is underpinned by the unique advantages they offer over cement-based grouts, particularly in the context of ancient building restoration. These advantages include moderate mechanical strength, water vapor permeability, and excellent compatibility with historical substrates [1,5,6,7]. However, the challenges associated with lime grouts, such as slow strength gain, low final strength, and notable shrinkage, require a meticulous exploration of their formulations [8,9].

In this context, in our study we undertake the comprehensive analysis of lime-based grout formulations, exploring the influence of mineral additions, chemical admixtures, and precise proportions, with a particular focus on the incorporation of metakaolin and natural volcanic pozzolan. The objective is to decipher the complex interactions among these components and understand their resulting effects on the properties of fresh lime grouts. To comprehend the trajectory of this investigation, we first delve into the contextual landscape of lime grout applications in historical conservation.

Historically, lime has played a pivotal role as a building material, admired for its enduring quality and versatile applications [10]. This tradition spans diverse cultures and epochs, with Vitruvius detailing the selection, calcination, and hydration processes of lime for mortar production [10,11]. Vicat’s contributions in the 19th century further solidified lime technology, emphasizing optimal slurry selection based on considerations such as injectability, durability, and mechanical properties [12].

Lime injection into masonry structures, an irreversible technique, has been widely employed for load capacity enhancement, the preservation of architectural surfaces, and structural continuity without morphological alterations [12,13]. The dominance of natural hydraulic lime in historical masonry grouting arises from its desirable attributes, including moderate strength, vapor permeability, and chemical compatibility with historical materials [4,13,14]. Given that the design of grouts hinges on substrate conditions, emphasizing factors such as penetrability, fluidity, mechanical strength, stability, and durability, the role of rheological studies becomes pivotal in lime slurry design [13].

The injection of grout is a consequential step in the consolidation and reinforcement of masonry structures and rock-excavated formations, offering viable solutions to environmental challenges [15,16,17,18,19]. The method ensures continuity, coherence, and strength without altering the morphology or structural system of the damaged structures [17,20]. However, as an irreversible application, meticulous design becomes imperative, particularly when dealing with historical structures [12,15].

Grout mixtures must be meticulously designed to achieve reliable performance characteristics such as injectability, strength, and durability [15,21,22]. Herein lies the core challenge; traditional cement-based grouts, despite possessing a higher strength and stiffness, are unsuitable for historical masonry due to issues such as salt crystallization, rigidity, and excessive strength [15,23]. In this context, NHL grouts emerge as a fitting alternative, aligning well with historical materials in terms of their mechanical, physical, mineralogical, and chemical characteristics [15,16].

The crucial parameters for achieving effective grout performance go beyond the mechanical aspects, encompassing fluidity, penetrability, water retention, stability, and bleeding. Achieving these properties often necessitates the incorporation of mineral additions and chemical admixtures, which influence the fresh-state characteristics of the grout [15,24]. Mineral additions, such as silica fume, brick dust, fly ash, and chemical admixtures, such as superplasticizer, viscosity modifier, and air-entraining agent, have been explored to enhance the performance of lime grouts [15,25]. Notable investigations have probed the effects of metakaolin on the properties of natural hydraulic lime-based grouts [8,15]. However, the comprehensive optimization of lime grout formulations for historical structure restoration remains an ongoing endeavor.

Lime-based grouts, while demonstrating potential in their compatibility with historical materials, pose challenges that require careful consideration. High water demand, resulting in volumetric instability, bleeding, and low mechanical strengths, poses significant hurdles [8,24,25,26,27,28]. In response, the study of high-speed mixing, superplasticiziers admixtures, and other types of mineral additions becomes paramount [24,25,26,29].

Research endeavors have explored the reinforcement of lime-based mortars with fibers, emphasizing the importance of the mixing procedure and the need to investigate suspension rheology, a dimension that remains relatively unexplored [8,25,26,27,28].

Metakaolin, an amorphous aluminosilicate obtained by calcining kaolinite, has gained traction as a mineral addition in lime grouts [24,26]. Its pozzolanic reactivity, contributing to enhanced mechanical properties and durability, positions it as a promising candidate for lime grout optimization [24,26]. Research by Vavričuk et al. emphasizes the potential benefits of using metakaolin in lime grouts, indicating improvements in compressive strength and durability [8]. Moreover, metakaolin’s ability to mitigate shrinkage and enhance workability underscores its multifaceted contribution to the rheological and mechanical properties of lime-based grouts [8,24].

Zhu et al. [30] found that the critical dosage of polycarboxylate-based superplasticizer in the NHL-2 type natural hydraulic lime pastes is 3%, beyond which fluidity experiences a gradual increase. A dosage range between 0% and 3% significantly reduces the yield point and plastic viscosity in NHL-2 pastes, changing the suspension behavior from plastic flow to Newtonian. This phenomenon is linked to the concentration of particles in NHL-2 suspensions, their flocculated structures, and the impact of the superplasticizer in breaking these flocs, improving particle dispersion and homogeneity.

A meticulous rheological study is fundamental, but rheology is not the only important factor; given the limited work carried out to date, the aim of this research is to demonstrate the significant importance of controlling rheological behavior based on rheological parameters: the yield point can serve as a control parameter for the application of injection slurries, influencing the slurry’s flow behavior and its ability to flow within porous media. The plastic viscosity is related to the speed at which the slurry will flow. Hence, both parameters can be utilized as control parameters in slurry pumping [21]. Finally, thixotropy governs the rate of structure acquisition in a suspension, determining its behavior at rest and its evolution over time.

This study explores the design of fluid lime-based grout formulations for heritage restoration, with a particular emphasis on the role of metakaolin and natural volcanic pozzolan, the latter being scarcely studied in compositions of these types of grouts. We have worked with NHL-5 type natural hydraulic lime pastes (these classifications refer to the strength of the lime, with NHL-2 being the weakest and NHL-5 being the strongest and the most hydraulic), which are characterized by having a higher degree of fineness and surface area, and poly-aril-ether-based superplasticizer, maintaining the same water–cement ratio and varying the admixture doses.

## 2. Materials and Methods

The quality and chemical composition of lime vary across different regions, emphasizing the importance of an initial study of its physical and chemical properties before its use in construction and restoration [31]. Natural hydraulic lime (NHL-5), natural volcanic pozzolan (NVP), and metakaolin (MK) were the powder materials used. A poly-aryl-ether-based polymer superplasticizer (MasterEase 5025, $\rho_{\mathrm{SP}}$ = 1058 kg/m${\;}^{3}$, dry residue 20%) [32], and deionized water constituted the remaining raw materials.

**Figure 1:**
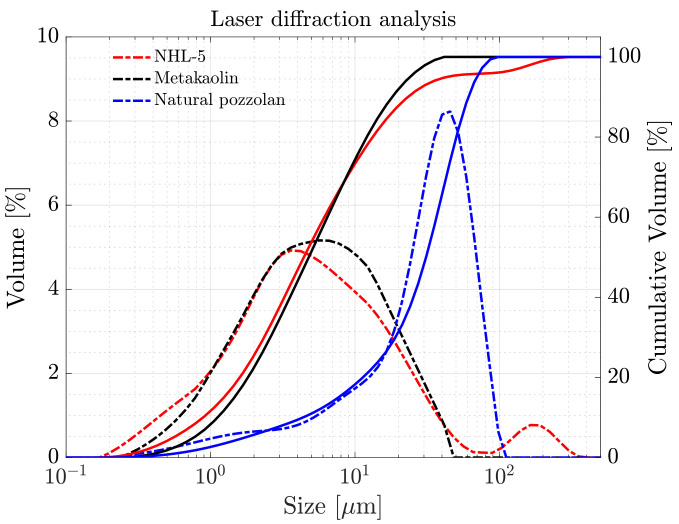
Particle size distribution of the three powder raw materials (solid lines correspond to cumulative volume, and the dashed lines correspond to volume).

**Figure 2:**
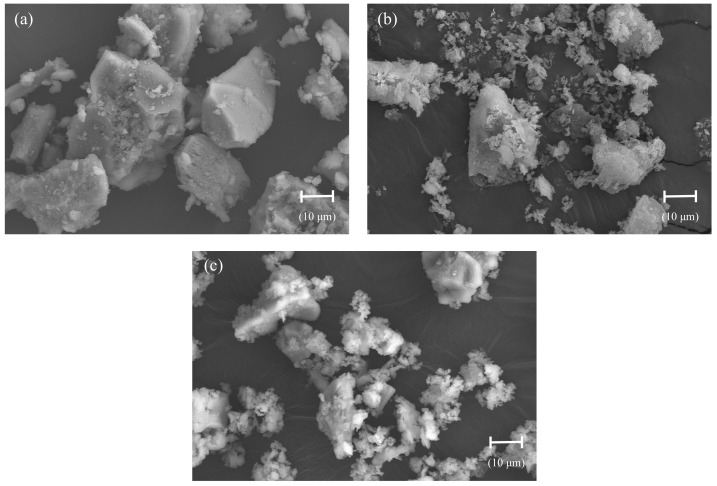
Back scattering electron microscopy (BSEM) images of particles: (**a**) NVP; (**b**) MK; (**c**) NHL-5.

The particle size distribution of the powder raw materials was determined using laser diffraction granulometry through an optical system Mastersizer 2000 (Malvern Panalytical, Malvern, UK) (Figure 1). BET surface area was established using a Quantonchrome TouchWin system (Quantachrome Instruments, Boynton Beach, FL, USA). Table 1 presents their physical properties (specific gravity, BET surface area, main particle sizes), and Figure 2 displays back scattering electron microscopy images, BSEM (Zeiss System (Oberkochen, Germany), with Oxford Instruments EDX (Abingdon, UK)) revealing the geometry, morphology, and textures of the powder materials. Table 2 includes the chemical composition (X-ray fluorescence, Malvern Panalytical, UK) of the powder raw materials.

Three types of pastes were prepared to study their flow behavior (Table 3): 100% NHL-5, 80% NHL-5 + 20% NVP, 80% NHL-5 + 20% MK, maintaining the water–binder ratio (w/b = 0.6) and varying the superplasticizer–binder ratio (SP/b = 1.0%, 1.5%, 2.0%, and 2.5%, referring to the percentage of the mass ratio between superplasticizer and binder).

The specific surface area influences the rheology of suspensions [13]. Superplasticizer admixtures decrease the yield point and plastic viscosity. Notably, a higher fineness of the binders requires a higher dose of superplasticizer for a given fluidity [33].

### 2.1. Mixing Protocol of the Suspensions

The suspensions were mixed in a beaker with a propeller-shaped blade, featuring a diameter smaller than that of the beaker to ensure thorough mixing. The space at the bottom, between the blade and the beaker’s bottom, was maintained at 5 mm.

For the suspension preparation, tap water was utilized, maintaining a stable environmental temperature. The mixing procedure was carefully controlled to ensure a representative and robust method. It proceeded as follows: water and superplasticizer were added and mixed at 400 rpm for 30 s; subsequently, the entire binder was slowly added and mixed at 400 rpm for 5 min. After incorporating all materials, an additional 5 min of mixing at 800 rpm was performed.

### 2.2. Rheometric Measurements

The influence of the characteristics of the measuring rheometer on the results must be taken into account. Since there is no universal testing procedure, the sample should be measured using the intended shearing procedure for its application [34]. The rheological properties of each suspension were analyzed using a rotational rheometer (Haake-Mars 40 Thermo Scientific, Waltham, MA, USA) equipped with a double cone–plate sensor (angle of 2° and diameter of 60 mm, with a truncated cone to prevent potential particle obstruction, and a gap of 0.088 mm) [35]. This geometry ensures that the sample completely fills the cavity, mimicking the flow conditions of a concentric cylinders sensor, and provides a uniform shear rate throughout the sample [36].

Following the mixing of each suspension, a sample was measured at a temperature of 25 ± 2 °C, testing it under shear rate control to prevent slippage between the suspension and the sensor walls. The rheological protocol, similar to those used in previous studies [37,38,39,40,41], involved a linear ramp upwards from 0 to 1000 s${\;}^{-1}$ over 180 s, 60 s at the maximum shear rate, and a similar linear ramp downwards from 1000 to 0 s${\;}^{-1}$. This protocol aims to establish steady flow properties in the suspensions [36] and achieve an equilibrium condition [42]. This approach ensures a uniform and completely de-agglomerated mixture. If the shear rate is not sufficiently high, the suspension may experience agglomeration and sedimentation [43]. The rheological measurements were carried out immediately following the mechanical mixing; however, it has been experimentally verified after conducting two consecutive measurements that the curves obtained in the second measurement were similar to the initial ones. So, the mechanical mixing protocol ensures the initial precision state of the suspensions.

## 3. Results and Discussion

### 3.1. Rheological Behavior of the Complete Flow Curves

In this investigation, we will differentiate between complete flow curves and the descending branches of the flow curves, which are composed of pairs of points representing shear stress and shear rate, τ-$\dot{\gamma }$. The flow curve provides information about the non-linear flow rheology of NHL-5-based suspensions in a range of shear rates, which is key to their behavior in processes like grouting.

Firstly, it should be noted that the particles in NHL-5 have a lower packing density within their solid granular structure, impacting the rheological behavior of the suspension [44]. This phenomenon, stemming from the morphology, texture, and particle size distribution, will be more or less pronounced depending on the characteristics of the added particles (NVP and MK). Morphological differences between both mineral additions are illustrated in Figure 2a,b. Table 1 emphasizes that both have a similar specific surface area value; however, regarding size distribution, the particles of NVP are significantly larger than those of MK.

**Figure 3:**
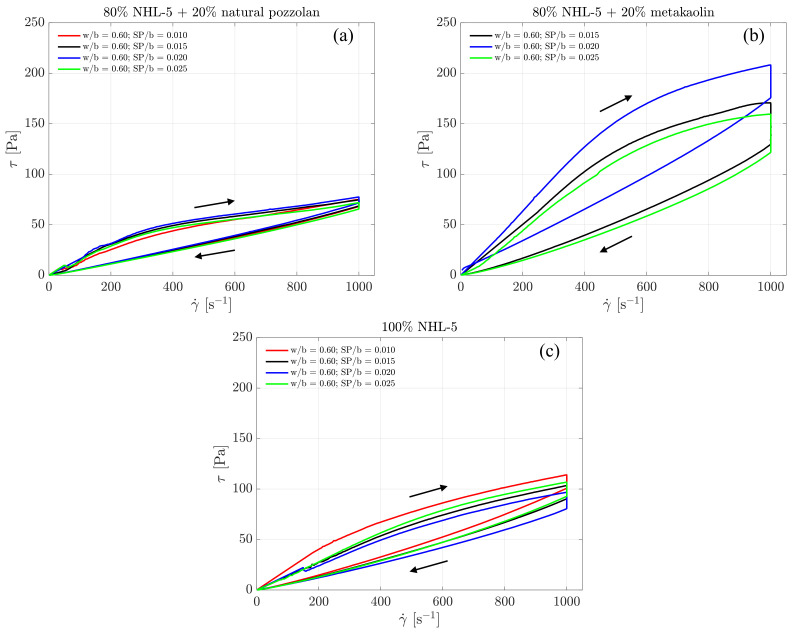
Complete flow curves for suspensions: (**a**) 80% NHL-5 + 20% NVP; (**b**) 80% NHL-5 + 20% MK; (**c**) 100% NHL-5. In all cases, the segments of the flow curves corresponding to the ascending branch of the rheological measurement protocol are above those corresponding to the descending branch of said protocol.

In Figure 3, the complete flow curves of the three suspension types are presented, each with varying doses of superplasticizer. It can be seen that the shear stress values at the maximum shear rate are around 75 and 100 Pa, for the suspensions of 80% NHL-5 + 20% NVP and 100% NHL-5, respectively, whereas for 80% NHL-5 + 20% MK suspension, values between 150–200 Pa are reached.

It is not customary to analyze the ascending branch of the flow curve in cementitious suspensions as it often exhibits irregular behavior; however, on this occasion, we will examine it because these branches do not show such irregularities. This is related to the high degree of structuration in the suspensions, which is a consequence of the presence of large particles and agglomerates and a lack of uniformity. Figure 3 demonstrates how, as the shear rate increases from rest, the corresponding shear stress also increases until it reaches the maximum value at the maximum shear rate.

**Figure 4:**
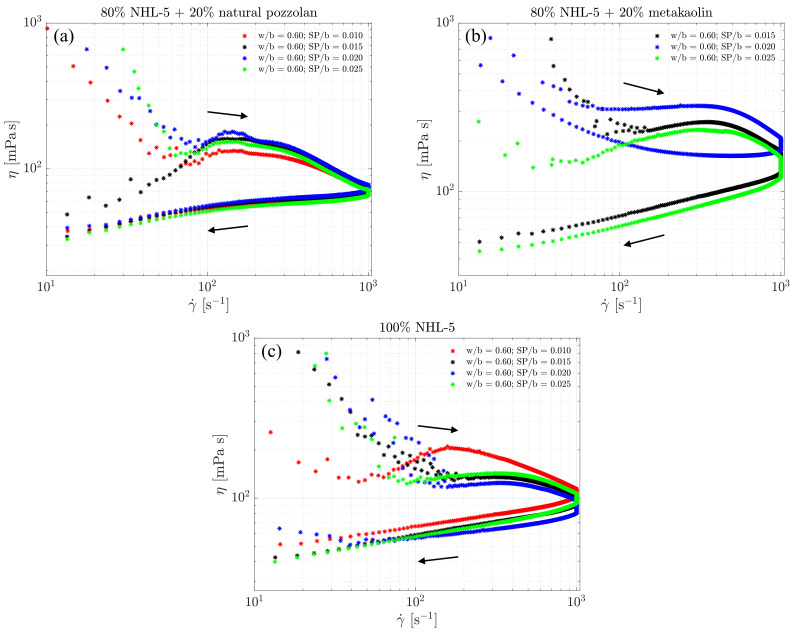
Complete viscosity curves for suspensions: (**a**) 80% NHL-5 + 20% NVP; (**b**) 80% NHL-5 + 20% MK; (**c**) 100% NHL-5.

This behavior is confirmed in Figure 4, where it can be observed that, as the shear rate increases from rest, the viscosity decreases from its initially high values. This is a result of the destruction of flocculated structures formed after the mixing of suspensions, leading to a more homogeneous suspension in terms of particle sizes. In a range of shear rate values below 100 s${\;}^{-1}$, viscosity starts to increase until it reaches the range of maximum values, beyond which it begins to decrease until it reaches the end of the initial ramp in the rheological measurement protocol.

However, the 80% NHL-5 + 20% MK suspension shows a different behaviour. With the maximum and minimum dosage of the admixture, the viscosity curves behave similarly. With the intermediate dosage of the admixture, the highest viscosity values are reached. Additionally, in this case, the descending segment of the curve shows an increase in viscosity as shear rate decreases. Since the rheological behavior of clay minerals is governed by surface charge [45], the viscosity is highly dependent on the interaction of MK particles with the superplasticizer admixture. The lower dosage produces an optimal effect in reducing viscosity due to strong particle repulsion; the intermediate dosage shows a change in behavior, increasing viscosity due to particle attraction. The higher dosage results in an admixture oversaturation, once again, causing suspension particles to experience strong repulsion and reducing viscosity.

### 3.2. Rheological Behavior of the Ascending Branches of the Flow Curves

If we differentiate between the flow curves for an ascending branch and a descending one, it can be verified that the Herschel–Bulkley model (Equation (1)) describes the non-linear rheological behavior of all the suspensions. As will be shown and analyzed in the following subsections, the fit is very good for all the cases in the ascending branches of the flow curves, and it is excellent for all the cases in the descending branches of the flow curves.

(1) $$\tau \;=\;\tau_{0}\;+\;k\;{\dot{\gamma }}^{n}$$

where,

τ: Shear stress at which the suspension is subjected;
$\tau_{0}$: Yield stress of the suspension;
*k*: Consistency coefficient of the suspension;
*n*: Flow index of the suspension;
$\dot{\gamma }$: Shear rate at which the suspension is subjected.

**Figure 5:**
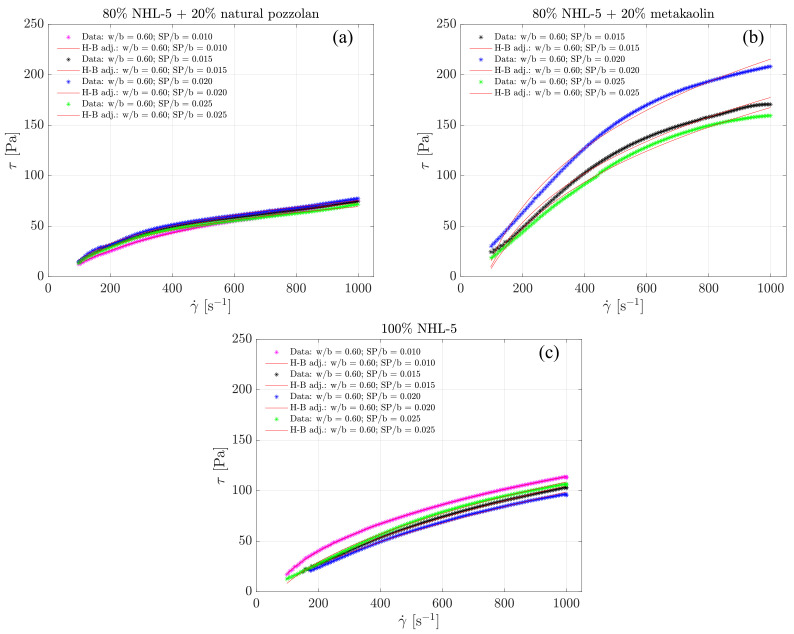
Data adjustment for descending branches of the flow curves of suspensions to the Herschel–Bulkley model: (**a**) 80% NHL-5 + 20% NVP; (**b**) 80% NHL-5 + 20% MK; (**c**) 100% NHL-5.

Figure 5 shows the ascending branches of the flow curves. A clear shear-thinning behavior in the three compositions and for all doses of superplasticizer admixture is observed in the shear rate range of 100–1000 s${\;}^{-1}$. Even before 100 s${\;}^{-1}$, the onset of this behavior is already observed. However, this shear rate range has been selected because it is from around 100 s${\;}^{-1}$ that the model allows for good adjustments.

On the other hand, the 80% NHL-5 + 20% MK composition presents very high viscosity values and increases in them compared to the other two compositions, particularly the one with a superplasticizer dosage SP/b = 0.020. Table 4 includes the values of the rheological parameters calculated by fitting the experimental data from the ascending branches of the flow curves to the Herschel–Bulkley model (range 100–1000) showing the shear-thinning behavior through *n* values, which are lower than 1.

### 3.3. Rheological Behavior of the Descending Branches of the Flow Curves

**Figure 6:**
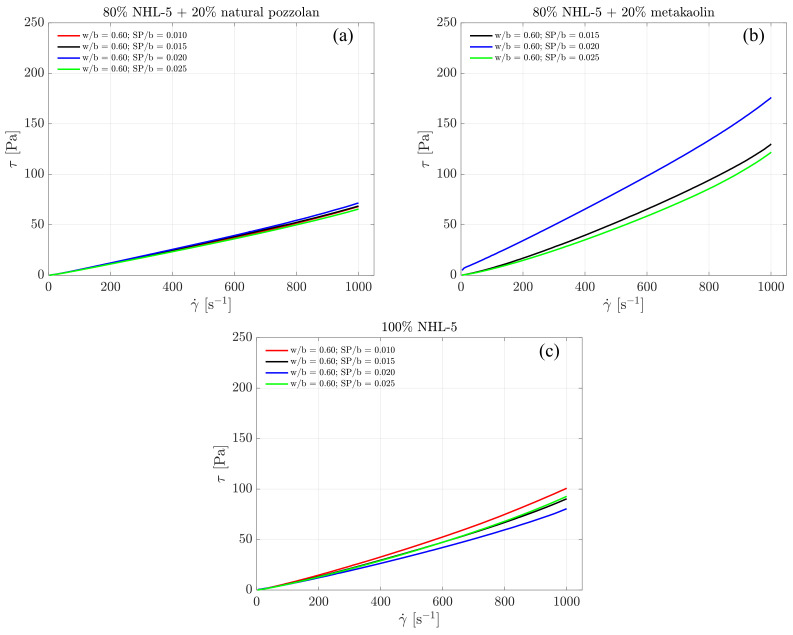
Descending branches of the flow curves of suspensions: (**a**) 80% NHL-5 + 20% NVP; (**b**) 80% NHL-5 + 20% MK; (**c**) 100% NHL-5.

Figure 6 illustrates the descending branches of the flow curves. Throughout this segment, the shear stress is notably lower, indicating the presence of thixotropic effects, as the apparent viscosity decreases with the flow duration. Ultimately, the descending flow curve concludes at the intersection with the ordinate axis, which can be considered as the dynamic yield point. In materials exhibiting thixotropic behavior, such as in this case, both the static yield point (measured on the ascending branch of the flow curve) and dynamic yield point (measured on the descending branch of the flow curve) depend on the previous flow history [46].

Under these conditions, the data from the descending branch are likely to be the closest to the effective flow curve of the material; that is, under conditions of steady-state flow [47]. Moreover, in the descending branches of the flow curves for all suspensions, the internal structure state reached after the first two ramps of the established rheological protocol allows for the erasure of shear history from the samples, enabling the analysis of each suspension under identical conditions.

It is worth noting that the previous statements about the stability of the suspensions are confirmed in this plot. The descending branch of 100% NHL-5 suspensions have small but real differences, with the lowest viscosity for a SP/b ratio of 0.020. In the 80% NHL-5 + 20% NVP suspension, the curves become practically coincident, thus demonstrating the increased stability of this formulation. On the contrary, the 80% NHL-5 + 20% MK suspensions have larger differences in viscosity, with the minimum value for the SP/b ratio of 0.025.

Considering that the surface area of NVP and MK is rather similar, the different effect in the viscosity when these powders are mixed with NHL-5 must be related to the particle size distribution. The NVP powder is significantly coarser that the other two, thus introducing a reduction in viscosity due to the bi-modality, since the replacement of fine by coarse particles helps to reduce the effective volume. In contrast, the MK powder has a similar size but a laminar shape that hinders homogenization.

Table 5 includes the values of the parameters $\tau_{0}$, *k*, and *n* calculated by fitting the experimental data from the descending branches of the flow curves to the Herschel–Bulkley model. In all cases, the suspensions exhibit shear-thickening behavior, as visualized in Figure 6, with values of *n* exponent greater than 1.

**Figure 7:**
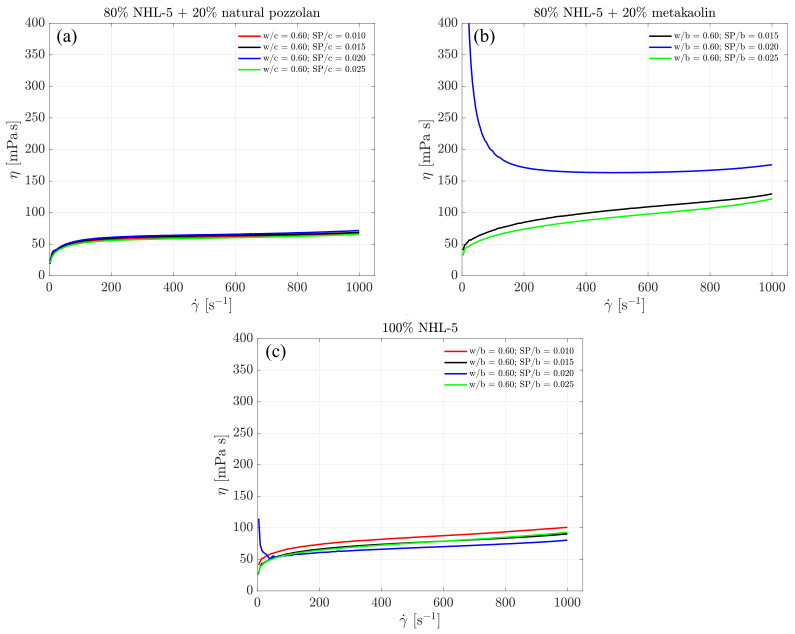
Viscosity of the descending branches of the flow curves of suspensions: (**a**) 80% NHL-5 + 20% NVP; (**b**) 80% NHL-5 + 20% MK; (**c**) 100% NHL-5.

As previously mentioned, it is of particular interest to analyze the rheological parameters associated with the descending flow curves. The design of slurry compositions to enhance injectability into porous media for specific applications is a key aspect that depends on intrinsic properties, as well as the nature and characteristics of the substrate [21]. In addition to the Herschel–Bulkley model’s index *n*, which provides information about the shear-thickening behavior of the suspension, the parameter *k* is associated with its viscosity. Figure 7 also displays the measured viscosity values for each suspension. Due to the rheological tests being conducted under shear rate control, observations at very low shear rates are not representative of the suspensions’ behavior in this region [35]. This implies that the dynamic yield stress cannot be precisely calculated.

The shear-thickening behavior in lime and lime-MK pastes, as observed by Sales et al. [45], is influenced by particle size, with smaller mean sizes causing a more substantial reduction in intensity, leading to a significant decrease in dynamic viscosity. Hydraulic lime suspensions exhibit yield points, and their non-Newtonian behavior is attributed to mechanisms in which shear stress orients particles in suspension, counteracting the random effects of Brownian motion [13]. The addition of a superplasticizer admixture induces a reduction in the yield point and viscosity, emphasizing its impact on the rheological properties of the suspension.

In all three compositions, viscosity values increase with the rise in shear rate. For the 80% NHL-5 + 20% NVP composition (Figure 7a), the viscosity remains nearly constant across all four doses of superplasticizer. In the case of the 100% NHL-5 composition (Figure 7c), lower viscosity values are achieved for the second-highest dose of superplasticizer (SP/b = 0.020), while higher values are observed for the lowest dose (SP/b = 0.010). As for the 80% NHL-5 + 20% MK composition (Figure 7b), notable discrepancies are observed in the viscosity curves. The highest values correspond to the intermediate dose of superplasticizer (SP/b = 0.020), while the smallest values are associated with the lowest dose (SP/b = 0.025). Furthermore, the intermediate dose (SP/b = 0.020) exhibits highly viscous behavior at low shear rates due to the characteristics of MK particles, leading to the formation of an internal structure in the suspension at shear rates below 200 s${\;}^{-1}$. A plausible reason for this can be attributed to the charge attraction–repulsion effect within the MK-superplasticizer system, as explained earlier. This shear rate range is quite high, requiring caution when selecting this superplasticizer dose for the 80% NHL-5 + 20% MK composition for applications involving shear rate ranges of 0–200 s${\;}^{-1}$.

**Figure 8:**
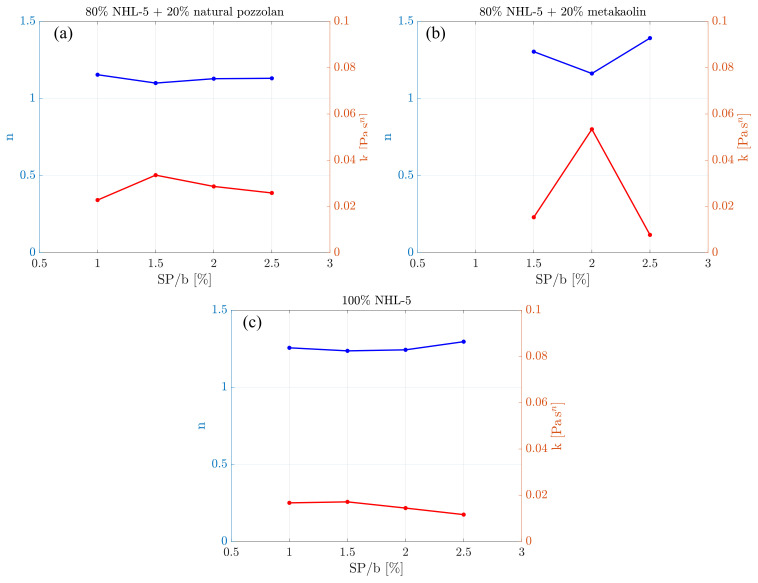
Relationship between *n* and *k* parameters of the Herschel–Bulkley model and superplasticizer dosage of suspensions: (**a**) 80% NHL-5 + 20% NVP; (**b**) 80% NHL-5 + 20% MK; (**c**) 100% NHL-5.

Shear-thickening is significantly influenced by the water-to-cement ratio, w/c, and the amount of superplasticizer admixture [41]. In this case, the w/c ratio remains constant, so the superplasticizer dosage defines the Herschel-Bulkley model parameters *n*. Figure 8 depicts the relationships between superplasticizer dosage and the Herschel–Bulkley model parameters *n* and *k*. Next, we are going to conduct an analysis of what is happening.

Regarding the parameter *n*, all three compositions exhibit shear-thickening behavior (*n*> 1). In both the 80% NHL-5 + 20% NVP and 100% NHL-5 compositions (Figure 8a,c), the values of *n* remain relatively constant for different superplasticizer dosages, with slightly lower values in the case of 80% NHL-5 + 20% NVP. However, the 80% NHL-5 + 20% MK composition (Figure 8b) reaches a minimum *n* value for the intermediate superplasticizer dosage (SP/b = 0.020).

Concerning the parameter *k* (related to viscosity), both the 80% NHL-5 + 20% NVP composition (Figure 8a) and the 100% NHL-5 composition (Figure 8c) show stable values across the entire range of superplasticizer dosages. However, the 80% NHL-5 + 20% MK composition (Figure 8b) exhibits greater variability in the behavior of this parameter. The minimum value of parameter *k* occurs for the highest superplasticizer dosage (SP/b = 0.025) for the 80% NHL-5 + 20% MK and 100% NHL-5 compositions, and for the 80% NHL-5 + 20% NVP composition at the lowest superplasticizer dosage (SP/b = 0.010).

The 80% NHL-5 + 20% NVP composition (Figure 8a) reaches a maximum value at the intermediate superplasticizer dosage SP/b = 0.015, corresponding to the minimum value of parameter *n*. The 100% NHL-5 composition (Figure 8c) attains the maximum value at the lowest superplasticizer dosage, SP/b = 0.010–0.015, also corresponding to the minimum value of parameter *n*. As for the 80% NHL-5 + 20% MK composition (Figure 8b), parameter *k* exhibits a clear maximum at the intermediate superplasticizer dosage, SP/b = 0.020, again corresponding to the minimum value of parameter *n*.

The incorporation of mineral additions plays a crucial role in influencing the rheology of suspensions. MK, regulated by surface charge, decreases the yield point and viscosity due to strong repulsive forces between particles [8,45]. In lime-based pastes, particularly those incorporating MK, several key observations and effects on rheological properties have been observed: regardless of MK content, a consistent reduction in the yield point and viscosity, a phenomenon attributed to factors such as volume fraction, shape, and surface charge of particles [45]. However, according to our results, the trend regarding incorporation is opposite, unlike with NVP, where the addition of a coarser phase to NHL, such as NVP, aids in compaction and reduces viscosity. However, MK has the same size as NHL, so both the platelet shape of MK and their surface charges come into play, along with their interaction with the additive. This reveals the complexity of interactions between materials in these systems and emphasizes the importance of conducting rheological studies when designing these types of suspensions based on their intended application.

### 3.4. Thixotropy

NHL-5 suspensions, inherently prone to particle agglomeration, exhibit a natural tendency towards structural breakdown. In our study, rheological tests revealed hysteresis cycles (Figure 6). The area of this loop in each curve represents the degree of structural buildup and breakdown that the corresponding suspension undergoes. It is the work required per time unit and volume unit to break some of the initially present linkages in the paste [48]. Thixotropy serves as an indicator of the degree of internal structure in the suspension, such as flocculation, early hydration, or broken polymeric chains [35,48,49,50,51], and is closely linked to shear-thickening in binder suspensions with superplasticizer admixture [43].

**Figure 9:**
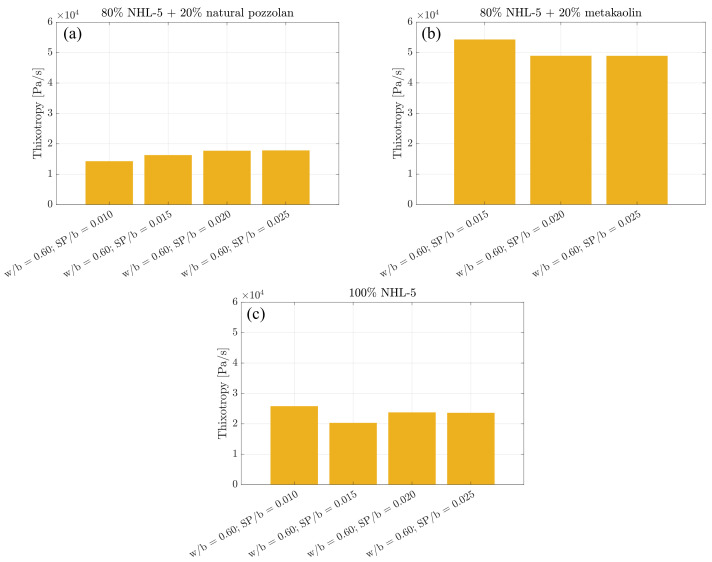
Thixotropy of suspensions: (**a**) 80% NHL-5 + 20% NVP; (**b**) 80% NHL-5 + 20% MK; (**c**) 100% NHL-5.

Thixotropy values remain quite consistent within each suspension type, regardless of the superplasticizer admixture content, with the lowest values obtained in the 80% NHL-5 + 20% NVP suspension (Figure 9a) and the highest in the 80% NHL-5 + 20% MK suspension (Figure 9b). This can be attributed to the ability of MK particles to generate more internal structures in the suspension compared to NHL-5 and natural pozzolan particles. This is in good agreement with the observed increase in viscosity.

In the 80% NHL-5 + 20% NVP suspension (Figure 9a), the lowest thixotropy value corresponds to the lowest superplasticizer dosage (SP/b = 0.010). In the 80% NHL-5 + 20% MK suspension (Figure 9b), the lowest thixotropy value corresponds to the highest superplasticizer dosages (SP/b = 0.020–0.025). In the 100% NHL-5 suspension (Figure 9c), the lowest thixotropy value corresponds to the intermediate superplasticizer dosage (SP/b = 0.015). In these cases, the electrostatic and steric repulsion mechanisms of the superplasticizer with the surface charges of the particles in each suspension come into play, requiring a higher or lower superplasticizer dosage depending on the characteristics of each suspension.

Lime pastes, similar to Portland cement pastes, exhibit a certain amount of de-structuring during flow, evolving their structure in both reversible and irreversible manners and being influenced by flow heterogeneities that arise throughout the test [47]. The same behavior is exhibited in lime pastes with mineral additions such as NVP and MK, as experimentally confirmed in this research.

### 3.5. Maximum Viscosity Ranges along the Flow Curve

**Figure 10:**
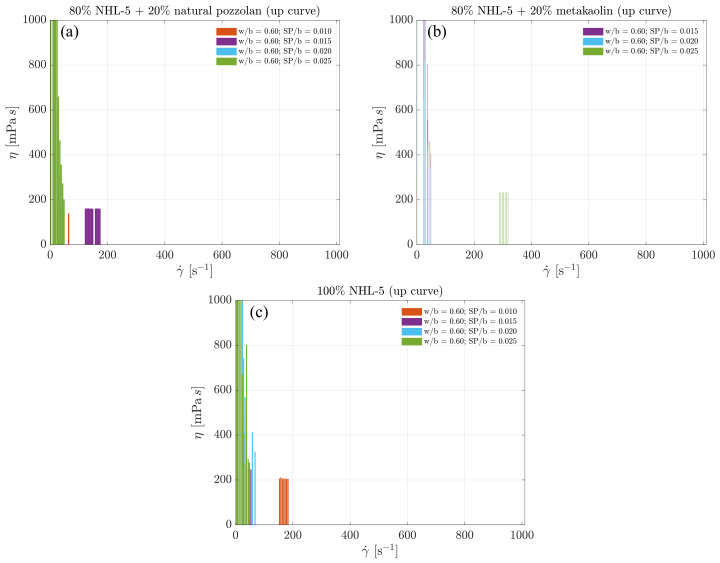
Maximum viscosity values measured in ascending branches of flow curves: (**a**) 80% NHL-5 + 20% NVP; (**b**) 80% NHL-5 + 20% MK; (**c**) 100% NHL-5.

Figure 10 show the maximum viscosity values measured from the three suspensions in the ascending branch of the flow curves. It can be observed that there are very high viscosity measurements in all three suspensions for all superplasticizer dosages. This is due to the destruction of floculated structures formed after mixing the suspensions, leading to a more homogeneous suspension in terms of particle sizes, as explained earlier.

However, the interesting part of Figure 10 lies in studying the viscosity values and shear rate ranges occurring under developed flow conditions. In the 80% NHL-5 + 20% NVP suspensions, the maximum viscosity values are slightly below 200 mPa s, occurring in the shear rate range of 150–200 s${\;}^{-1}$ and corresponding to dosages of SP/b = 0.015 (Figure 10a). In the 80% NHL-5 + 20% MK suspensions, the maximum viscosity values are slightly above 200 mPa s, occurring around shear rates of 300 s${\;}^{-1}$ and corresponding to dosages of SP/b = 0.025 (Figure 10b). In the 100% NHL-5 suspensions, the maximum viscosity values are equal to 200 mPa s, occurring in the shear rate range of 175–200 s${\;}^{-1}$ and corresponding to dosages of SP/b = 0.010 (Figure 10c). The measured maximum viscosity values are very close in the three suspensions and occur in similar shear rate ranges. The main difference lies in the superplasticizer dosage at which it occurs. This effect may be related to the colloidal properties of solid particles in suspension: depending on the morphology, size, composition, and surface electric charges, maximum values are reached in the ascending flow curve. Higher viscosity values associated with larger shear rate intervals correspond to 80% NHL-5 + 20% MK, which is due to the characteristics and properties of MK particles. Lower viscosity values associated with smaller shear rate intervals correspond to 80% NHL-5 + 20% NVP, a consequence of the characteristics and properties of NVP particles, which have a lesser flocculation effect when added to NHL-5. These results are consistent with intermediate measurements obtained in 100% NHL-5 suspensions.

It is evident that the highest viscosity measurements in the descending branches of the flow curves of the three suspensions occur at the maximum shear rate used in the test. The highest viscosity values correspond to lower dosages of superplasticizer (80% NHL-5 + 20% MK and 100% NHL-5). The 80% NHL-5 + 20% NVP suspension exhibits the lowest maximum viscosity values, while the 80% NHL-5 + 20% MK suspension shows the highest.

Finally, it is interesting to note that, once again, very high viscosity values appear at very low shear rates in the descending branch of the flow curve for the 80% NHL-5 + 20% MK suspension. This may be explained by the characteristics of MK particles associated with their physicochemical properties (shape, size, morphology, composition, electric charges). Maximum viscosity values at very low shear rates also appear in the 100% NHL-5 suspension, although they are very low and may be a consequence of reflocculation processes. The 80% NHL-5 + 20% NVP suspension does not exhibit high viscosity values at very low shear rates.

The design and processing of this type of suspension, depending on its application, are closely linked to the study of rheology. Once the water–binder relationship, which conditions the mechanical strength, is established, determining the optimal superplasticizer dosage through rheological parameters (flow point, plastic viscosity, and thixotropy), as well as adjusting a model that allows estimating the evolution of these parameters, represents a crucial step in the use of NHL-5 and pozzolanic materials for heritage conservation and sustainable construction.

### 3.6. Effects on Lime-Based Systems of the Interaction between Materials

Interactions occurring among the various components in each of the suspensions lead to a series of synergistic and antagonistic effects. Among the former, it is noteworthy that the variation in particle size distribution due to the inclusion of additives results in a higher degree of packing, benefiting both viscosity and the reduction in pore size and, therefore, weak zones in terms of the mechanical response in the hardened state of the suspension. This is reflected in Figure 1, where it can be observed that both NHL-5 and MK have a similar particle size distribution; however, NVP presents a wider particle size range, and when combined with NHL-5, it produces a system with a higher degree of packing, which is reflected in the flow curves (Figure 3a, Figure 6a and Figure 8a), viscous behavior (Figure 4a, Figure 7a and Figure 8a), and thixotropy of the suspension (Figure 9a). Moreover, the high pozzolanic reactivity of both NVP and MK represents a potential source for the formation of C-S-H and C-S-(A)-H gels in a system with such a high content of portlandite, implying an improvement in the mechanical response, especially in the medium and long term.

As for antagonistic effects, the high surface area of NVP and MK (Table 1) implies a high affinity for water adsorption, which results in a higher demand for superplasticizer additive to achieve the desired fluidity level according to the processing technique (in this case, we are focusing on slurry injection). In addition, the electrostatic and steric repulsion mechanisms of the superplasticizer interact with the surface charges of the particles in the suspensions, especially with the metakaolin particles. This results in a variable superplasticizer dosage depending on the characteristics of the clayey particles, and the optimal value must be identified for the suspension to exhibit the desired rheological behavior (Figure 8b).

### 3.7. Implications for Applications: Injectability and Strength Development

In processes such as slurry injections, having information on rheological parameters, such as the yield point and flow stress, and their evolution with shear rate, is crucial [52]. The former provides information about the onset of flow, while the latter informs us about the speed of this flow. The nonlinear behavior of this type of suspension influences the efficiency of slurry injection processes, which must be controlled as they can lead to both poor executions and equipment failures. The measurement of thixotropy determines the rate of internal structure formation in the suspension, which impacts the injection process (rest, movement, and shear rates).

The characteristics of mechanical mixing and the application process through injection, as well as the evolution of the rheological properties of the suspension, will determine the mechanical properties and their development. This is because their design and behavior in the fresh state are closely linked to their behavior in the hardened state in this type of suspension. More deflocculation and uniformity in the microstructure implies lower porosity, leading to a reduction in weak zones and, consequently, greater, and a better development of mechanical strengths.

## 4. Conclusions

In this research, a systematic study is presented for optimizing rheological properties of NHL-5 injection grouts for sustainable construction and heritage conservation. Specifically, this paper studies the rheology of NHL-5 suspensions with and without replacement by NVP and MK. Three types of NHL-5-based suspensions were studied with different compositions in terms of the typology of binders and the dose of superplasticizer. Using a double cone–plate rotational rheometer, and following an appropriate rheological protocol, the suspensions were subjected to a high shear rate (up to 1000 s${\;}^{-1}$) to study their flow curves.

This comprehensive rheological investigation delves into the complex flow behavior of NHL-based suspensions augmented with mineral additions such as volcanic pozzolan and MK. The study strategically differentiates between complete flow curves and their descending branches, offering nuanced insights crucial for applications like grouting. The morphological and distribution variances of mineral particles profoundly impact the flow behavior, with implications for packing densities within the granular structure.

Distinct rheological responses are observed based on the morphology and texture, and the particle size distribution of mineral particles. Despite similar specific surface areas, differences in particle size contribute to unique rheological behavior. The investigation categorizes flow behavior into shear-thickening, with shear stress increasing proportionally with shear rate until a maximum is reached. Superplasticizer dosage is identified as a critical factor influencing viscosity, particularly at low shear rates, indicating its role in the breakdown of floculated structures formed after suspension mixing.

Descending flow curves reveal thixotropic behavior, characterized by lower shear stress compared to the ascending branch. The Herschel–Bulkley model effectively captures this non-linear behavior, providing valuable insights into the rheological parameters associated with the descending flow curves. Thixotropy, integral to understanding structural evolution, displays variations across different compositions, offering valuable information for tailored suspension designs.

Shear-thickening behavior, assessed using Herschel–Bulkley parameters, exhibits dependency on superplasticizer dosage. Intriguingly, compositions containing MK display an opposite trend in shear-thickening behavior compared to those with natural pozzolan. This unexpected observation underlines the intricate interplay of material interactions within these systems.

The analysis of thixotropy reveals varying degrees of structural buildup and breakdown, influenced by superplasticizer dosage and particle characteristics. This comprehensive study provides a foundation for understanding the impact of composition design on the internal structure of suspensions.

Further exploration of maximum viscosity ranges along the flow curve elucidates the conditions and dosages influencing viscosity. Our research highlights the importance of considering superplasticizer dosage and particle characteristics in optimizing the rheological properties of NHL-5-based suspensions. This knowledge is crucial for achieving specific application requirements, particularly in terms of their injectability into porous media.

In summary, this research contributes valuable insights about the intricate rheological nuances of NHL-5-based suspensions with mineral additions. By unraveling the complexities associated with mineral particle characteristics and superplasticizer dosage, the study provides a foundation for designing suspensions tailored to specific applications. This understanding is pivotal for advancements in sustainable construction materials, offering a roadmap for future research and development in this domain.

## Figures and Tables

**Table 1:** Properties and particle sizes of powder raw materials.

	NHL-5	NVP	MK
Specific gravity	2.80	3.15	2.66
Surface area (BET) [m${\;}^{2}$/g]	5.4	19.1	17.2
D${\;}_{90}$ [ μm]	22.5	60.1	18.0
D${\;}_{50}$ [ μm]	4.5	31.1	4.8
D${\;}_{10}$ [ μm]	0.9	4.1	1.1

**Table 2:** Chemical composition of NHL-5, NVP, and MK.

	Chemical Composition (%)		
	**NHL-5**	**NVP**	**MK**
Na${\;}_{2}$O	0.348	3.932	0.100
MgO	8.346	10.982	0.230
Al${\;}_{2}$O${\;}_{3}$	4.053	13.850	41.439
SiO${\;}_{2}$	13.650	43.349	52.848
P${\;}_{2}$O${\;}_{5}$	0.060	0.591	0.142
SO${\;}_{3}$	1.524	0.022	0.031
Cl	0.020	0.027	–
K${\;}_{2}$O	1.090	1.469	1.328
CaO	53.897	9.145	0.035
TiO${\;}_{2}$	0.227	2.886	0.563
V${\;}_{2}$O${\;}_{5}$	0.041	–	0.008
Cr${\;}_{2}$O${\;}_{3}$	0.014	0.053	0.009
MnO	0.040	0.144	0.006
Fe${\;}_{2}$O${\;}_{3}$	1.864	9.496	1.425
Co${\;}_{3}$O${\;}_{4}$	–	0.014	–
NiO	0.017	0.029	0.012
CuO	–	0.008	0.003
ZnO	0.004	0.012	0.006
Ga${\;}_{2}$O${\;}_{3}$	–	–	0.004
Rb${\;}_{2}$O	0.005	0.005	0.013
SrO	0.219	0.089	0.016
Y${\;}_{2}$O${\;}_{3}$	–	0.003	0.003
ZrO${\;}_{2}$	–	0.048	0.026
Nb${\;}_{2}$O${\;}_{5}$	–	0.006	0.002
BaO	–	0.041	0.013
CeO${\;}_{2}$	–	0.053	0.044
Pr${\;}_{2}$O${\;}_{3}$	–	–	0.009
PbO	–	–	0.015
CO${\;}_{2}$	14.580	3.747	1.669

**Table 3:** Mix design composition of NHL-5-based suspensions (w/b = 0.6).

	Raw Materials [kg/m${\;}^{3}$]					
**Denomination**	SP/b	**NHL-5**	**NVP**	**MK**	**Water**	**Superplasticizer**
100 NHL-5	0.010	1034.7	0	0	620.8	10.4
	0.015	1029.7	0	0	617.8	15.5
	0.020	1024.7	0	0	614.8	20.5
	0.025	1019.8	0	0	611.9	25.5
80 NHL-5 + 20 NVP	0.010	834.6	208.7	0	626.0	10.4
	0.015	830.5	207.6	0	622.9	15.8
	0.020	826.5	206.6	0	619.9	20.7
	0.025	822.5	205.6	0	616.8	25.7
80 NHL-5 + 20 MK	0.015	820.5	0	205.1	615.4	15.4
	0.020	816.5	0	204.1	612.4	20.4
	0.025	812.6	0	203.2	609.4	25.4

**Table 4:** Parameters of the Herschel–Bulkley model calculated for the suspensions in ascending branches of the flow curves (w/b = 0.6); 95% confidence bounds are shown in parentheses.

Denomination	SP/b	$\dot{\gamma }$ [s${\;}^{-1}$]	$\tau_{0}$ [Pa]	*k* [Pa s${\;}^{n}$]	*n*	$R_{\mathrm{adj}}^{2}$
100% NHL-5	0.010	100–1000	–	19.6 (18.7, 20.5)	0.323 (0.318, 0.328)	0.999
	0.015		–	3.0 (2.4, 3.6)	0.546 (0.520, 0.573)	0.998
	0.020		–	1.7 (1.2, 2.3)	0.610 (0.569, 0.650)	0.994
	0.025		–	8.5 (6.7, 10.3)	0.425 (0.400, 0.450)	0.998
80% NHL-5 + 20% NVP	0.010	100–1000	–	9.4 (7.7, 11.1)	0.355 (0.335, 0.376)	0.998
	0.015		–	155.8 (104.0, 207.5)	0.094 (0.074, 0.114)	0.998
	0.020		–	171.3 (95.0, 247.7)	0.089 (0.063, 0.115)	0.997
	0.025		–	177.7 (93.9, 261.5)	0.084 (0.057, 0.110)	0.997
80% NHL-5 + 20% MK	0.010	100–1000	–	115.3 (63.2, 167.5)	0.198 (0.157, 0.239)	0.993
	0.020		–	204.7 (100.3, 309.1)	0.164 (0.121, 0.206)	0.993
	0.025		–	68.0 (38.9, 97.2)	0.245 (0.202, 0.287)	0.993

**Table 5:** Parameters of the Herschel–Bulkley model calculated for the suspensions in descending branches of the flow curves (w/b = 0.6); 95% confidence bounds are shown in parentheses.

Denomination	SP/b	$\dot{\gamma }$ [s${\;}^{-1}$]	$\tau_{0}$ [Pa]	*k* [Pa s${\;}^{n}$]	*n*	$R_{\mathrm{adj}}^{2}$
100% NHL-5	0.010	0–1000	1.305 (1.054, 1.557)	0.017 (0.016, 0.018)	1.256 (1.247, 1.265)	0.999
	0.015		0.897 (0.675, 1.119)	0.017 (0.016, 0.018)	1.236 (1.227, 1.245)	0.999
	0.020		1.297 (1.088, 1.505)	0.015 (0.014, 0.015)	1.243 (1.233, 1.253)	0.999
	0.025		1.277 (1.044, 1.509)	0.012 (0.011, 0.012)	1.296 (1.286, 1.306)	0.999
80% NHL-5 + 20% NVP	0.010	0–1000	0.714 (0.528, 0.900)	0.023 (0.021, 0.024)	1.155 (1.146, 1.164)	0.999
	0.015		0.228 (0.059, 0.397)	0.034 (0.032, 0.035)	1.101 (1.093, 1.108)	0.999
	0.020		0.501 (0.304, 0.698)	0.029 (0.027, 0.030)	1.129 (1.120, 1.138)	0.999
	0.025		0.454 (0.291, 0.617)	0.026 (0.024, 0.027)	1.131 (1.124, 1.139)	0.999
80% NHL-5 + 20% MK	0.010	0–1000	1.164 (0.851, 1.476)	0.015 (0.014, 0.016)	1.304 (1.295, 1.314)	0.999
	0.020		8.298 (7.779, 8.816)	0.053 (0.050, 0.057)	1.162 (1.152, 1.173)	0.999
	0.025		1.850 (1.429, 2.270)	0.008 (0.007, 0.009)	1.392 (1.377, 1.407)	0.999

## Data Availability

Data will be made available on request.
